# Of the Helmholtz Club, South-Californian seedbed for visual and cognitive neuroscience, and its patron Francis Crick

**DOI:** 10.1016/j.shpsc.2013.11.012

**Published:** 2014-03

**Authors:** Christine Aicardi

**Affiliations:** Department of Science and Technology Studies, University College London, Gower Street, London WC1E 6BT, United Kingdom

**Keywords:** Cognitive neuroscience, Vision neuroscience, Interdisciplinary networks, Francis Crick, Semi-institutions, Scientific and intellectual movements

## Abstract

Taking up the view that semi-institutional gatherings such as clubs, societies, research schools, have been instrumental in creating sheltered spaces from which many a 20th-century project-driven interdisciplinary research programme could develop and become established within the institutions of science, the paper explores the history of one such gathering from its inception in the early 1980s into the 2000s, the Helmholtz Club, which brought together scientists from such various research fields as neuroanatomy, neurophysiology, psychophysics, computer science and engineering, who all had an interest in the study of the visual system and of higher cognitive functions relying on visual perception such as visual consciousness. It argues that British molecular biologist turned South Californian neuroscientist Francis Crick had an early and lasting influence over the Helmholtz Club of which he was a founding pillar, and that from its inception, the club served as a constitutive element in his long-term plans for a neuroscience of vision and of cognition. Further, it argues that in this role, the Helmholtz Club served many purposes, the primary of which was to be a social forum for interdisciplinary discussion, where ‘discussion’ was not mere talk but was imbued with an epistemic value and as such, carefully cultivated. Finally, it questions what counts as ‘doing science’ and in turn, definitions of success and failure—and provides some material evidence towards re-appraising the successfulness of Crick’s contribution to the neurosciences.

## Highlights

• Question what counts as doing science and reconsider standards of failure and success.
• Crick’s contribution to neurosciences was significant contrary to the received view.
• Crick promoted his plans for neuroscience through patronage, networking and influence.
• The uses of a ‘mere’ scientific club can cut across many dimensions of science.

## 1 Introduction

“My guess is that history will tell, many years from now, that a lot of the key, seminal ideas in the area of vision primarily, but about other parts of the brain too, came out of discussions held at Helmholtz meetings. That’s something you probably won’t hear about because the meetings aren’t published. New ideas happen in small groups, not in big meetings. Big meetings are where the results of ideas are presented.” (Sejnowski, 2000, p. 330)

“I’ve learned a lot from everybody I’ve run into. I’m shameless about adopting ideas from people. … My most intense learning experience—has been this thing called the Helmholtz Club. I don’t know if you’ve heard of it. … There’s maybe twenty people there. I never miss one. I have somebody take my class because it lands right on top of my class. I do it anyway because it’s just too important to miss.” (Mead, 2000, p. 138)

In the early 1990s, Pnina Abir-Am suggested that historians should focus on “intermediary units of sociohistorical analysis … collaborative patterns of various duration, such as research schools, circles, clubs, and other informal gatherings, which combine a definable social structure with coordinated research programs”, on the grounds that they were indispensable for understanding the history of many interdisciplinary fields in twentieth-century science (Abir-Am, 1991, p. 342). Abir-am’s suggestion can be taken up in two complementary ways, each pointing at a different body of relevant scholarship. The first is to act on the presupposition that she has a valid point, and engage into empirical case studies framed accordingly. And indeed, there is a distinguished if not dominant tradition in history of science, running over the past four decades or so, of sociological historians who have chosen this path and produced richly detailed evidence in favour of said presupposition.^1^ The second is to ask why Abir-Am may have a valid point, and try and theorize it. Attempting to find patterns in the scatter of empirical case studies and to build them into a systematic approach is a more recent strand of research, rooted into social studies of science, political sociology, and social movements studies. It has largely sprung from the observation that nascent interdisciplinary fields jostling at the margins of institutional disciplines to try and, or fail to, transform mere prospective visions into established scientific and intellectual programmes may have much in common, in their dynamics and lifecycles, with social movements. The tentative outline of a broad theory of scientific and intellectual movements has notably been proposed by Scott Frickel and Neil Gross in the 2000s, and has fostered recent scholarship broadly aimed at re-generating the political sociology of science.^2^

The present paper endorses the view that semi-institutional gatherings of the kind that Abir-Am was bringing to attention will prove to have been instrumental in creating sheltered spaces, metaphorical greenhouses, into which many a 20th-century project-driven interdisciplinary research programme could take root, and eventually flourish into a fully-fledged ‘big science’ endeavour. It explores the history of one such gathering from its inception in the early 1980s into the 2000s, the Helmholtz Club, which brought together scientists from such various research fields as neuroanatomy, neurophysiology, psychophysics, computer science and engineering, who all had an interest in the study of the visual system and of higher cognitive functions relying on visual perception such as visual consciousness.

Taken on its own, the present paper falls into the isolated empirical case studies category; however, this microstudy fits within a larger project revisiting the life of the late Francis Crick, British molecular biologist and geneticist of double-helix fame who turned neuroscientist in the late 1970s. Borrowing heavily from Frickel’s and Gross’s ideas of scientific and intellectual movements and from social movements literature, the theoretical approach, which it is not within the scope of the paper to detail, adopts the analytical lens of ‘scientific activism’ in an attempt at making sense of Crick’s dispersed scientific career as a consistent whole.^3^ A premise of this approach is that ‘doing science’ is a highly social pursuit that can occur in an array of mundane spaces beside the laboratory bench or the seclusion of the office, and that involves many kinds of activities beside the canonical ones: attending clubs and seminars, sitting on boardroom meetings, lobbying funders, etc. The fact that in their memory work, a majority of scientists, Crick among them, would not insist much on these aspects of their careers in comparison to the actual content of their research, like for instance they would not insist on the time spent writing grant proposals, is not surprising—few scientists see such activities as being a significant part, even a part at all, of their ‘doing science’. Yet it is part of the historian’s job to question the scientists’ memory work, and question how much these side activities are also ‘doing science’. This has further implications, as questioning what counts as ‘doing science’ in turn questions what counts as ‘success’ and ‘failure’ in science.^4^ In the case of Crick, the received wisdom about his long engagement with the neurosciences, as measured according to standards where what counts as ‘success’ are the intellectual or experimental achievements of the individual scientist, is that it was rather a failure since he did not make any significant breakthrough on the major problems he set out to solve. This is materially reflected in the two published book-length biographies of Crick, by popular science writer Matt Ridley and academic historian Robert Olby.^5^ Both accounts are largely framed by a traditional understanding of what counts as doing science and both devote around four times as much space to Crick’s Cambridge-based molecular biology and genetics research as they do to his neuroscience research in San Diego, despite the comparable periods of time spent by Crick at the MRC Laboratory for Molecular Biology on the one hand and at the Salk Institute for Biological Studies on the other hand—roughly twenty-five years in each case.

The present paper, as part of the chain of evidence that the overall project is assembling, argues that Crick had an early and lasting influence over the Helmholtz Club of which he was a founding pillar, and that from its inception, the club served as a constitutive element in his long-term plans for a neuroscience of vision and of cognition. Further, it argues that in this role, the Helmholtz Club served many purposes, the primary of which was to be a social forum for interdisciplinary discussion, where ‘discussion’ was not mere talk but was imbued with an epistemic value and as such, carefully cultivated. Finally, it provides some material evidence towards re-appraising the successfulness of Crick’s contribution to the neurosciences.

## 2 A vision ‘tie club’

In the spring of 1982, V.S. Ramachandran was a postdoc in the psychophysics of vision at UCI (University of California Irvine), working on perceptual illusions. Francis Crick was J.W. Kieckhefer Distinguished Research Professor at the Salk Institute for Biological Studies in San Diego, where he had moved permanently in 1976 and begun a new career in the neurosciences that he was to pursue to the end of his life. Crick’s focus was then on the visual system, and he was keen to witness Ramachandran’s experiments, which he had heard of through his Cambridge-based collaborator Graeme Mitchison who was corresponding with Ramachandran. Crick and Ramachandran met briefly on the occasion of a two-day workshop at UCI organised in early May 1982 by physicist-turned-theoretical-neurobiologist Gordon Shaw, with who Ramachandran was doing experimental work on apparent motion (Shaw & Ramachandran, 1982). Like many other young scientists who got to attract Crick’s interest and meet with him, Ramachandran was quite taken with his enthusiasm and approachability. Ramachandran remembers that after Crick’s hurried tour of his lab, sitting later in the cafeteria with Shaw, he was recounting the whole episode and at some point asked Shaw, wouldn’t it be wonderful to start a club, similar in spirit to the RNA Tie Club, which would do for vision what the RNA Tie Club reputedly had done for early molecular biology but would hold actual meetings rather than just be a correspondence club, and to involve Crick in it? Shaw apparently got very excited by the prospect. The idea for the Helmholtz Club had hatched.^6^

At the UCI workshop, Crick and Ramachandran had settled on a date for Crick to pay the latter’s lab a more thorough visit, late May or early June. According to Crick’s agenda for the period, the day was June 1, 1982, as this is the only day with a trip to UCI marked down.^7^ Ramachandran must have broached the club idea during the visit, and Crick must have liked it and made some suggestions, because thanking him for his visit a few days later, Ramachandran wrote:

“Vision seems to be in a state of confusion similar to pre-DNA molecular biology. What we need is more people with a compulsive urge to unmake mysteries—which is why I found it so inspiring talking to you!”

And added in post-scriptum:

“Gordon [Shaw] and I will draw up a list for a vision ‘tie club’.”^8^

And so they did. Ramachandran and Shaw established a preliminary list of possible people, with the view of having a diverse array of research fields represented like neurophysiology, anatomy, the psychophysics of perception and theoretical computer modelling. They approached Joaquin Fuster, neurophysiologist from UCLA, and John Allman, neurobiologist at Caltech. Ramachandran had been a postdoc and an associate in Allman’s lab prior to his going to UCI, and it was at a party organised by Allman in December 1980 that Ramachandran had first been introduced to Crick and “enjoyed chatting with [him] about Visual Perception”.^9^ Both Allman and Fuster became actively involved from the start, and well into the 2000s when the Helmholtz Club started winding down. Soon, Ramachandran and Shaw drove down to the Salk Institute to discuss the list with Crick, who added some more names that he thought well worth enrolling.^10^

As with any club, there was the question of membership to settle. The restricted membership formula that was thought out in the first instance was to limit the number of members to about fifteen, with each member allowed to bring one guest to any session.^10^ In keeping with its ‘British club’ inspiration, the Helmholtz Club had a Secretary, who was in charge of running the operation.^10^ There are conflicting testimonies as to who was playing that role, Ramachandran or Shaw, during the first few years. What is for certain is that Terrence Sejnowski took over the job when he joined the Salk Institute in 1988 and has remained Secretary in title to this day.^11^ After this handover, membership apparently became more informal. According to neurophysiologist and neurosurgeon Joseph Bogen who became a regular member from around 1990 until 2003 when his failing health prevented him to further participate, “[t]he principal value of speaking at the Helmholtz Club was being added by Terry Sejnowski to his list of invitees” (Bogen, 2006, p. 112). Sejnowski agrees that the number of members nominally on the club roster, i.e. his circulation list, rose in time to probably around forty-five names. Yet it does not appear to have impacted significantly the average size of the group present at any session. Although former participants remember that numbers could fluctuate a lot from about ten people to thirty plus, at which times Room C at UCI University Club where the meetings invariably took place felt crowded, the goal according to Sejnowski was to have between ten and twenty people in any session so that they could all sit around a table at dinner, and they averaged about fifteen. The ‘one member-one guest’ principle remained in place, as it was felt that it helped keep things moving by providing a constant input of ‘fresh blood’ attending in the form of students, colleagues or visitors:

“… if you have this small group that meets over and over again, after a while you get too self-absorbed. … They have their own views because they keep going around in circles, talking to each other, instead of getting input from other people outside … Bringing in outsiders … and immersing them in this environment, that really helped open it up.”^12^

Beside the question of membership, there was the question of the name. It was decided collectively at the first meeting to name the ‘vision tie club’ after nineteenth-century German physicist and physiologist Hermann Von Helmholtz. They deemed it appropriate to pick as their patron a figure celebrated as the ‘father’ not only of modern theories of visual perception but also of electrophysiology—in 1850, Helmholtz was the first to measure the velocity of transmission of ‘animal electricity’ in the nerve, showing in the process that it travelled much too slowly to be a current of Voltaic electricity as previously thought (Clarke & Jacyna, 1987, p. 187; Crick, 1994, pp. 14, 96; Ramachandran, 1990).^10^

The club’s first meeting was probably held 22 September 1982. The corresponding entry in Crick’s 1982 appointment book agrees with the place where the Helmholtz Club would be held, the University Club at UCI; with the weekday on which it would be held, Tuesday; and with the timeframe that Ramachandran and Allman remember.^13^ Fittingly, the meeting is unspecified, as picking a name for the club was part of the agenda.^10^ An early-Autumn meeting also corresponded with the start of the academic year. The availability of grant money to fund the club from June 15, 1982 (see Section 3) is another element in favour of the first meeting taking place right after the summer. Crick reported that for 1982–83, the group met “roughly once a month (about eight times a year).”^14^

Ramachandran, Shaw, Crick, Allman and Fuster must have attended this first meeting. Although there is no knowing with certainty who constituted the original membership, the following list of individuals cannot be far off the mark, for they were all ‘locals’ at the time the club was launched, their involvement with the Helmholtz Club is documented at some point, and their early attendance either has received direct confirmation or I have presumed it based on their having given talks in the club’s first year: neurophysiologist Richard Andersen, the Salk Institute; vision neuroscientist Irv Biederman, University of Southern California (USC); experimental psychologist of visual perception Walter Gogel, UC Santa Barbara (UCSB); Donald Hoffman, computational psychology and cognitive science, UC Irvine (UCI); theoretical and computational biophysicist John Hopfield, Caltech; Simon LeVay, neurobiology and neurophysiology of the visual system, the Salk Institute; Don MacLeod, psychophysics of vision, UC San Diego (UCSD); John Schlag, neurobiology of vision, UC Los Angeles (UCLA); Madeleine Schlag-Rey, cognitive neuroscience of vision, UCLA; David van Essen, neurobiologist of visual processes, Caltech; Norman Weinberger, neurobiology of learning and memory, UCI; ex-molecular biologist turned cognitive scientist David Zipser, UCSD. Over time, new members joined, others left; some came only occasionally; some were active for a few years but then would drop out as they moved away from Southern California—this was typically the case with postdocs introduced to the club by regular members, who would then attend for a few meetings. Some would also stop coming because they changed research interests. There was a bit of a ‘fading away’ pattern apparently and commenting on it, Allman and Mead underlined that Helmholtz Club meetings took the whole day, about ten days a year:

“This is a significant time commitment. … You didn’t do it unless you were really committed.”^15^

However, a core group of members remained actively committed from the moment they started attending club meetings and for much longer than average. Among those were neurophilosopher Patricia Churchland, who moved to UCSD in 1984^16^; Thomas Albright, neuroscientist of the visual system, who was recruited by the Salk Institute in 1987^17^; computational neurobiologist and systems neuroscientist Terry Sejnowski, who joined the Salk Institute faculty beginning of 1989 and took over as Secretary of the club the year before^12^; computer scientist and engineer Carver Mead, Caltech, who gave a talk in April 1989 so must have joined around that time if not before (he does not remember when) and became a regular;^18^ neurophysiologist and neurosurgeon Joseph Bogen, UCI, who recalled that he had been invited to give a talk and from then on became a regular too, dating it back to 1995 (Bogen, 2006, p. 111) although he may have had the year wrong as in fact he first gave a Helmholtz Club lecture in December 1989;^19^ and Allman, Fuster, Shaw and Crick.^20^

For more than twenty years after its September 1982 launch, the Helmholtz Club would invariably follow the same pattern of meeting about once a month through the academic year, on a Tuesday (in over a hundred inventoried meetings, only two did not take place on a Tuesday), at UCI University Club.^10^ The routine did not vary one bit:

“As usual, lunch will be available in the dining room from 1:00 to 2:00 p.m. We will then meet from 2:00 to 6:00 in Room ‘C’. Dinner will follow the talks.”^21^

As for the organisation of the sessions, the format was to have two speakers, one a guest and the other a member of the club, giving lectures on related issues or topics, with enough time for discussion after each talk (which often overrun until around 6:30), and a break in-between with coffee, tea, doughnuts and cookies.^22^

The decision to pick Irvine as meeting place was motivated by thoughtful geographical considerations. The club organisers wishing to involve researchers located in as many South-Californian institutions as possible (UCI, UCSD, UCLA, UCSB, Caltech, the Salk Institute, USC), Irvine was conveniently central. In the very early days of the club, they gave a try at holding a meeting in an outside restaurant, by the sea in Dana Point about twenty miles south of Irvine. Ramachandran recalls that it had a great ocean view. They decided that the food was excellent but the location did not work well as it was not central enough, and they settled once and for all at UCI University Club.^23^ As for dinner, it was a leisurely affair with wine and lively discussion, generally taking place at the Pinot Provence in Costa Mesa, close to Irvine.^24^

## 3 Francis Crick’s long-term plans for a neuroscience of vision and visual consciousness

A question that must have arisen quite soon after Ramachandran and Shaw started bouncing around the idea of a ‘vision tie club’ was how they were going to fund it. Not that it was expected to be particularly expensive, but still, if it was to hold regular meetings involving an active group of members belonging to various South Californian institutions, they needed funds to book a seminar room on a regular basis as well as to reimburse the travel, and eventually lunch and dinner, expenses of the club members. And if they were to invite speakers, they needed more funds to cover their travel and living expenses as well. At this point, historical serendipity came into play. A few months before, in January 1982, Crick had submitted a five-year grant proposal to the System Development Foundation (SDF). The grant was approved by the board of the SDF on 15 June 1982, a three-year grant with renewal possibilities, at $300,000 per annum, and Crick could start drawing on the funds immediately. Furthermore, the purpose of the grant was to support the build-up of a combined experimental and theoretical approach to understanding the higher nervous system with a focus on the visual system, and it included a Visitors Program (one in three lines of budget approved by the SDF out of the six constituting the original proposal), which aim was to “promote useful interactions between theorists, and between theorists and experimentalists”. This Visitors Program fell under three heads, one of which was:

“i. Seminars. I expect one seminar a month for nine months each year.”^25^

The Helmholtz Club had not been written in Crick’s original grant proposal in January 1982, yet the idea of a ‘vision tie club’ came at an opportune moment to get funded through the SDF award by embodying the seminar series that was part of its Visitors Program. The simple fact that this conjunction of events did occur certainly adds weight to the view that Crick had much input in the project as it took shape and then unfolded.

It is impossible to assess the extent to which fitting Ramachandran’s and Shaw’s nascent project within Crick’s SDF-funded research programme moulded the former in the process precisely because the club was still in the very early stages of its conception at the time, when ideas matured while being bounced around and were therefore hard to credit to a particular source. Nonetheless, I would like to make a couple of remarks. First, the RNA Tie Club was a correspondence forum where ideas and experimental results were circulated between its members, and if it was indeed such a forum that Ramachandran and Shaw had in mind, it would not have involved inviting external speakers to give lectures but rather having members present their unfolding ideas and experimental trials to each other; whereas a seminar series that was part of a Visitors Program implied invited speakers giving lectures, which was the case with the Helmholtz Club.

Second, in his grant proposal, Crick explained that his plan was to focus mainly on the visual system, and to some extent on other cortical functions involved in the detailed processing of highly complex information. Crick further explained that the approach he intended to pursue would “concern itself with the detailed wiring and activity inside the brain”, combining the theoretical framework of the then newly-hatched computational approach exemplified by David Marr’s ideas (which were about to be published posthumously in his landmark book *Vision*) Marr (1982) with “a lot of evidence from neuroanatomy, neurophysiology, evolved potentials, positive emission tomography (PET) and so on … This might be called the Integrated Computational Approach … The hope is that it will be possible to tie together theory, psychophysics, neurophysiology, neuroanatomy and molecular biology in at least a few specially favorable cases.” Crick added that he would thus need “frequent contact with experimental groups working in this area.” As he recalls in his autobiography, having chosen to focus on the visual system due to practical considerations he thought that he could “help to build bridges between the various scientific disciplines, all of which studied the brain from one point of view or another” (Crick, 1988, p. 152). In the context of this plan of his, the Visitors Program, and within it the seminar series that the Helmholtz Club was to embody, “would promote useful interactions between theorists, and between theorists and experimentalists.”^26^ This precisely matched the interdisciplinary mix of both theoretically and experimentally-bent researchers, focusing on the visual system and related cognitive functions, that the Helmholtz Club would aim at bringing together.

Such an approach to the visual system and more generally to neuroscience was not mainstream at the time. Computational approaches themselves were still at the budding stage. The term ‘computational neuroscience’ had not yet been defined—this would be the explicit purpose of the Symposium on Computational Neuroscience held in Carmel, California, on June 5 1987, which Eric L. Schwartz started planning in 1985 at the invitation of none other than the SDF. Many of the then active Helmholtz Club members (Michael Arbib, Pat Churchland, Christof Koch, Carver Mead, Terry Sejnowski, David van Essen, David Zipser) and some of its occasional visitors (Max Cynader, Misha Mahowald, David Robinson) participated in the symposium and contributed chapters to its accompanying book (Schwartz, 1990, pp. v–xiii). Crick was seeking to promote a distinctive research framework for the neuroscience of vision and of the higher cognitive functions and this, not as a one off in the context of the SDF grant, but consistently over the years, as he firmly believed in the value of studying large brain systems beside how individual neural cells operate, of linking various levels of study from the molecular to the systems and cognitive levels:

“Francis, I think, recognised the importance of bringing neuroscience into the field of vision, because vision up until that point was dominated by psychophysics, which are experiments … at the behavioural level. And Hubel and Wiesel,^27^ that was a revolution because they really were teaching us a lot about how the brain represents the visual world, and I think Francis saw that as a way to try to build a deeper understanding of vision by connecting the cellular level with the behavioural level. And I think it has been very successful in that regard over the years. It was critical, I think, a turning point, because there was new people coming into the field [from many different areas of science], who didn’t have resources and funding. And the fact that Francis was able to … promote what we were doing made a huge impact in terms of funding agencies.”^12^

Coming back to the SDF, funding new areas of research was indeed their goal—“not the things that were already well established but new things … that could maybe have an impact down the road.” 12 Without entering into the details of a complex and eventful corporate history, it is worth making a short aside to situate the Santa Monica-based System Development Foundation. It was a not-for-profit corporation which had originated in the 1950s as a division of the RAND Corporation dedicated to programming for the computers in the US Air Force SAGE air defence system. RAND had first spawned his System Development Division as the not-for-profit System Development Corporation (SDC) in 1956, until a change in Air Force policy in 1966 amounted to SDC being thrown into the commercial market, leading its Trustees, by 1969, to seek a change towards profit-making status. Following negotiations with the Air Force, the Internal Revenue Services (IRS) and the California State General Attorney, it was agreed that, in essence, the original SDC would be split into a profit-making corporation retaining the SDC name which would inherit the business (facilities, operations and contracts) and a not-for-profit foundation, SDF, which would retain the Board of Trustees of the original SDC and receive a large majority share of the for-profit corporation stock. Moreover, since the not-for-profit SDC had been tax-exempt for over ten years and made its money through Government contracts, it was agreed that the SDF would sell its SDC stock, and once paid four million dollars pledged to the Treasury, distribute the rest of the proceeds to the American taxpayers by making grants to tax-exempt institutions that sponsored research in fields somewhat related to the original SDC activities. This process took longer than initially planned and SDC stock was not floated on the open market as it had originally been intended. Instead, in 1980, Burroughs acquired SDC as a wholly owned subsidiary, with the merger becoming effective in January 1981. It appears that as owner of about two-third of SDC stock at the time of the merger, the SDF realised roughly $66 million dollars; once their tax obligations settled, this would have left them with some $62 million to part with (Baum, 1981, pp. 26–29, 279–281; Dobbins, 1993, pp. 2–3).

The idea was to distribute all SDF funds over a finite period of time; by 1984, half of its resources had been committed and it closed down in 1988. In order to develop its grant-making program, in 1981 SDF Trustees appointed as Director of Programs Charles S. Smith, formerly with the Department of Energy in Washington DC. They also consulted with outside experts, who were invited to give presentations to the Board to help them formulate funding guidelines. Among them was Crick, who despite his relative newness to the broad domain he was consulted about, carried the weight of his Nobel-laureate prestige. His presentation to the Board on October 20, 1981 detailed his comprehensive ‘Integrated Computational Approach’. In December 1981, the funding guidelines were issued by the Board, which defined the areas in which the SDF was to support research: “principles of information science, computational linguistics and speech, symbolic mathematics, robotics, non-Von Neumann computation, neurosciences, human-machine interface, and computer music.” Meanwhile, Smith got the green light to solicit proposals actively (Dobbins, 1993, pp. 4–5). The guidelines apparently also stipulated that the foundation would favour novel research, which “at the edge of current scientific work has little opportunity to get government funding.”^28^

Predictably, Crick and the Salk Institute were among Smith’s first round of solicitation; he came to visit in December 1981, accompanied by SDF Board member Augustus B. Kinzel, who had in the past been the first president and chief executive officer of the Salk Institute and was still one of its Trustees (as well as one of Caltech). Salk Institute President Frederic de Hoffman, in his cover letter draft for Crick’s grant proposal, addressed to Smith and dated January 15, 1982, extended his thanks on his and Crick’s behalf for allowing them to prepare a proposal on “a computational approach to an understanding of the higher nervous system” and emphasized that:

“The possibility of funding the proposed project by the Systems Development Foundation comes at a most opportune time. This would enable Dr. Crick to have a real impact on the ‘new’ neurosciences.”^28^

Also among the early grantees whose award must have been approved alongside Crick’s by the SDF Board at their June 15, 1982 meeting was Sejnowski, who wrote to Crick on July 5, 1982 that he had “recently received a two-year grant of $345,000 from the System Development Foundation to study motor computation in the central nervous system using the high-resolution 2-doxyglucose technique” and that he intended “to study the pattern of activity in the motor system of trained animals, and in particular to study the functional organization of motor cortex and the cerebellum. An image processing system will be used to collect and analyse the data.”^29^ Mead and Zipser were two other Helmholtz Club grantees. Confirming the at-the-margins nature of research funded by the SDF, Mead commented: “They supported our stuff for about six or seven years, when no government agency would touch it because it was way too early and too speculative and too controversial.” (Mead, 1996, p. 25)

Returning to Crick’s SDF grant and the Helmholtz Club, the latter was explicitly identified with the Visitors Program’s seminar series of the former in Crick’s annual reports to the SDF. This is how it appeared in the report for the year July 1, 1982–June 30, 1983, first report in the series:

“II. THE HELMHOLTZ CLUB

This is a small group of scientists working in Southern California with a special interest in the visual system. Members meet at Irvine (because of its convenient location) roughly once a month (about eight times a year) from lunch time through dinner time. Speakers are either members or visitors from elsewhere, often those who are part of my visitors program. There is ample time for discussion, both formal and informal. I [have] used a small amount of my funds to help support this activity.”^30^

Under the Visitors Program, Crick invited researchers for visits which in the first year “ranged in duration from two days to nine weeks.” In the four subsequent years, he had about ten visitors each year, but they tended to stay for shorter visits—he had found “very demanding” the experience of having visitors around for about forty-three weeks in total (with some overlaps) in the first year.^30^ Among Crick’s invited researchers under the Visitors Program who gave guest lectures at the Helmholtz Club, one finds visual neuroscientist Horace Barlow, neurophysiologist David Hubel, neuropsychologist Richard Gregory, experimental psychologist Anne Treisman.^31^

For the second year, from July 1, 1983 to June 30, 1984, Crick reported to the SDF:

“II. THE HELMHOLTZ CLUB

This continues to be very successful. In fact we now have to worry about keeping down the numbers attending. Experience shows that we cannot have more than two speakers for a 2:00–6:00pm session, because the discussion is usually extensive. The Club has met eight times since last July.”^32^

He continued on a more laconic mode in the three subsequent reports. Third year, 1984–85: “This continues to be very useful. The Club has met nine times since last July.” Fourth year, 1985–86: “This continues to flourish. It has met six times since last July.” Fifth year, 1986–87: “This continues actively though the attendance fluctuates widely from one meeting to the next. It has met nine times during the past fiscal year.”^33^Eventually the SDF money ran out. When precisely is not entirely clear, as the documents kept by Crick in relation to the SDF grant give only a partial view of said grant’s lifecycle. Yet Crick’s initial proposal was for a five-year grant and although it was first awarded for three years with renewal possibilities, Crick sent annual reports to the SDF until June 30, 1987, so it appears that this was the date at which it expired and that the grant was indeed extended to five years—hence Crick must have received about $1.5 million in total from the SDF. The grant could not have gone on much longer, though, since the SDF closed down a year later, on 30 June 1988. After that, the Salk Institute stepped in and covered all expenses, including lunch and dinner.^12^ This may initially have been envisaged as a temporary solution—in August 1988, as Sejnowski was preparing to move from Baltimore to San Diego in the Autumn and take over as Secretary of the club, he wrote to Crick that he would “take care of drafting a proposal for further support”, adding that the MacArthur Foundation might be interested^34^—but eventually the Salk Institute went on funding the Helmholtz Club for over twenty years, until after Crick’s death in 2004.

There is little doubt that convincing the Salk Institute management to start sponsoring the Helmholtz Club, and to go on when no other sources of funding materialised, was mostly Crick’s doing. Back in 1987, beside Crick, the only other scientist at the Salk Institute who was a Helmholtz Club regular was Simon LeVay. Richard Andersen had been another early member but left that year. LeVay, a neuroanatomist working on visual cortex in animals, did not have Crick’s seniority, nor was he full professor. Moreover, personal tragedy was about to disrupt his life and lead him to change fields of research by the early 1990s. It is a mark of the influence that Crick wielded at the Salk Institute, that despite chronic fund-raising problems (Bigelow, 1995), they would agree to thus bankroll the Helmholtz Club for over two decades. It is also a mark of the importance that the Helmholtz Club held for Crick, beside the time commitment that it represented, that he would campaign for its funding.

At this point in the story, having explained how Crick first came to shoulder the responsibility for funding the Helmholtz Club through his SDF grant, and how moulding it to fit within his pre-established SDF project may have altered the original idea of a ‘vision tie club’ that had dawned on Ramachandran that day of Spring 1982 in the cafeteria at UCI, we should consider to what extent the Helmholtz Club eventually emulated the RNA Tie Club. Granted, the RNA Tie Club, initiated by theoretical physicist, cosmologist and cryptologist George Gamow in 1954, was remembered by some of its illustrious participants, and in the molecular biology community, for being a forum where important ideas and experimental results were circulated that related to the nature of the genetic code.^35^ Yet it was narrowly focused on ‘cracking’ the genetic code, rather short-lived, and its members were scattered between England, South Africa and both the coasts of the United States, corresponding rather than actually meeting. Thus it may have been more an ideal of the RNA Tie Club that Ramachandran had in mind than the real thing—by his own account, it was certainly the English tradition of gentlemen getting together and discussing some scientific topic, going all the way back to the early days of the Royal Society. The idea of having a Secretary followed from the same tradition.^10^ With hindsight, the Helmholtz Club had actually more in common with the Hardy Club that Crick actively attended in Cambridge during the 1950s than with the RNA Tie Club.

In terms of group size, the Helmholtz Club was very comparable to either the Hardy Club, which counted about twenty members (de Chadarevian, 2002, p. 91) or the RNA Tie Club, which counted exactly twenty members each designated after one of the twenty amino acids. There is no reason to believe that Crick held sway over the size of any of these three clubs. But the consummate social animal that he was, serial group-member and seminar-goer,^36^ would have had an experience-informed appreciation of group dynamics and of what constituted a ‘good’ group size; and Crick would certainly have objected to significantly enlarged attendance, like he apparently did in the case of the Cognitive Science seminar that in the late 1980s was instituted at UC San Diego to replace the weekly PDP seminar he used to attend assiduously from 1982 to 1987 (the PDP group had counted about fifteen regular members)^37^:

“… it was one of those things that Francis sort of predicted, he said, it is going to get too big and it won’t be good anymore. And that is what happened. … There was a discussion but it was just the standard seminar. It wasn’t this interactive group that it had been.”^38^

Another similarity between the three clubs, their membership was by invitation only, and they bore names that would speak only to the happy few involved. We have seen how the ‘vision tie club’ project came to be named after Hermann von Helmholtz. The RNA Tie Club was named after the then still mysterious ribonucleic acid and had a special tie with a green and yellow RNA helix emblazoned on it. The Hardy Club, “a small, somewhat exclusive biophysics club”, founded in 1949 in Cambridge, took its name from the late William Bate Hardy, Cambridge zoologist turned physical chemist, and it seems that the purpose of having such a sibylline name was to not make it obvious that it was a biophysics club and offend people who had not been asked in (de Chadarevian, 2002, p. 91). Indeed, such clubs used to be a fixture of British academic life and during his time in Cambridge, beside the Hardy Club, Crick had also been a member of two physics clubs, the Kapitza Club and the Δ^2^V Club.^39^

Where the Helmholtz Club had more in common with the Hardy Club than with the RNA Tie Club is in terms of format and conviviality. The general format followed by the Helmholtz Club consisted in having a guest and a member give a lecture each with extensive discussion time, preceded by lunch and followed by dinner, and meetings took place about once a month during the academic year. The Hardy Club was an evening club that met once or twice a term; one of its members, occasionally a guest, gave a presentation followed by discussion; and the speaker was customarily treated to an early dinner prior to his talk and often, according to Crick, quite a lot to drink (he recalls that James Watson was no exception when he came to present the model of the DNA structure, and was “slightly bleary-eyed”). In contrast, the Helmholtz Club treated its guests to lunch, tea and dinner, but I have had no mention of lunchtime drinking before the talks (although wine was a fixture at dinner as I mentioned earlier)—it may have been a bit early in the day, and these were different times in a different country (Crick, 1988; de Chadarevian, 2002, pp. 91, 78–79).

These similitudes, and especially the closeness in format between the Helmholtz Club and the Hardy Club, may not come as a surprise. Crick had much input into the Helmholtz Club as it shaped up, and while the club was meant by its instigators to take inspiration from a British model, among the club founders Crick was the only one who could actually draw on a past ‘British clubber’ experience—in particular that of the Hardy Club, which ran successfully for a full decade from 1949 until the late 1950s or early 1960s, and which by Crick’s own recognition had been very useful to him as a forum of exchange between different fields of research sharing common interests (de Chadarevian, 2002, pp. 91–93). One detail is quite telling: the first invited speaker of the Helmholtz Club, on October 19, 1982—this was the first official meeting of the ‘named’ club after the September 1982 launch meeting—was Cambridge neurophysiologist Horace Barlow, another original member of the Hardy Club, who according to Crick was the one who had first introduced him to vision neurophysiology when around 1953 he had presented his research on the frog’s retina to his Hardy Club fellows (Crick, 1988, p. 148).^40^

## 4 A multipurpose enterprise

Having explored the birth of the Helmholtz Club, its membership, its modus operandi and its funding, it remains to be seen which purposes it may have served, starting with that of being a social forum for interdisciplinary discussion—and here I will argue that discussion, as it was construed at the Helmholtz Club, carefully crafted and perfected to something of an art, was imbued with an epistemic value.

First, it should be emphasized that ‘discussion’ was indeed the primary aim of the Helmholtz Club. Nurturing discussion was what the format of the sessions was precisely geared towards: a warm-up over lunch, then around two hours set aside for each speaker with the idea of having as much as one hour for questions after the talk—this is unusual enough—and finally for those who still felt up to it, dinner, arranged so that conversations could go on at length. But more than that: “ … the speaker was, in a sense, just an excuse for us to have a discussion. Literally. … The purpose of the questions was the purpose of the club. … The speaker was there just to help … move the discussion.”^12^ It was not unusual for speakers to be so bombarded with questions that they could not get past the first few slides, occasionally the very first slide, of their presentation, making their experience of the Helmholtz Club quite memorable.^41^

The presence of the speaker would thus help focus the discussion; yet this would not have prevented it from veering off into a specialist argument that only a couple of people were interested in and from which most of the participants, due to the widely interdisciplinary nature of the club, would have been excluded. There is a consensus that Sejnowski helped a lot; he was a skilled moderator of free-wheeling discussion, who helped maintain balance and focus. According to Albright, Mead played the role of the naïve-yet-shrewd outsider: “Carver was always asking questions … He is not a neuroscientist but he would … zoom in on what the most important issue was and he certainly understood what good neuroscience was.” Crick too had an important role in leading discussions and re-directing conversations. It seems he had a knack for popping up with interesting questions even when what was talked about was obscure and boring, and for keeping things focused on what was thought to be important for both the group and the research field. According to Allman and Mead, “he would be your perfect graduate student in a sense”, who asked these searching and penetrating questions that made a topic accessible and kept the discussion on the right intellectual plane, when it could easily have sunk into narrowly specialized technicalities.^42^

But there was a quality to discussion at Helmholtz meetings, which closely depended on two other traits of the club. First, as a grass-root, co-opted and rather confidential ‘semi-institution’, it sat somewhat in-between the public and private spheres. Second, as its members ritually met for a near-entire day once a month during the academic year, and further interactions and collaborations developed between them, inter-personal trust relationships grew and deepened.^43^ Combined, these two characteristics allowed for the Helmholtz Club to become a place where speculative ideas could be bounced around without typical academic inhibition and the sentence of public peer judgement—a place that belonged to what social geographer Hester Parr has described as “a ‘geography of license’, where to behave ‘differently’ is acceptable…” (Parr, 2000, p. 227). Sejnowski thus echoed this particular quality of the Helmholtz Club: “There were people who were saying things in a small group that they wouldn’t say in public, but that they really thought; their real views, not what they were going to write in a paper, not what they were going to tell in a public talk.”^12^ Albright commented along similar lines: “People certainly let down their inhibitions and they would just talk about anything … And we all knew one another. So it was sort of very informal, and collegial … It was easy to ask questions, and easy for people to approach material that they didn’t understand, and not feel any inhibitions about it.”^17^

The result was that for Sejnowski, discussions at Helmholtz meetings—carefully cultivated interdisciplinary group-thinking between hand-picked individuals from an array of interested fields—served a higher cognitive purpose. It was an epistemic tool aimed at triggering biological intuitions:

“… in biology, experience and intuition play a much bigger role, maybe not so much bigger but it plays a different role, than in physics, which is much more formalised and very well developed in terms of mathematics and applications to the real world. And … how do you get a biological intuition? … You can read all the books, but that doesn’t really help, because those things just tell you facts … You have to have something based on experience that gives you intuition. … Leo Szilard … when he got into biology he said it had changed his lifestyle, because the thing he loved most was soaking in a hot bath tub, thinking about physics problems, for hours. The trouble was, he said, when you started thinking about biology problems, you’d have to get out of the bath every couple of minutes to look up a fact. So that is what you are up against in biology. Even before you get off the ground, you have to know an enormous amount. And the people sitting around that table [at the Helmholtz Club] were encyclopedias … and if you are part of the club, you have access to their experience, right? It is like being in a very high level … masterclass. Like in a masterclass in music, what happens is you bring someone like Pablo Casals, and … all the other aspiring musicians, … they have already mastered the technical part, but the part that he brings in is the feeling, is musicianship … that was passed onto him from his mentor. … Something is going on there which is way beyond anything that words can capture. It is really … at a very high cognitive conceptual level. And that was what was going on at the Helmholtz Club.”^12^

The epistemic benefit of bringing together embodied experience drawn from various fields of research to trigger ‘biological intuition’ in the neuroscience of vision was felt if not conceptualized by other Helmholtz Club members, and exploited. A pattern developed, where invitations to speak at the Helmholtz Club involved much more for guests than just giving a talk at a Helmholtz meeting; their visit became the pretext for a ‘tour’ of the various Southern California institutions represented at the club, which augmented dramatically the possibilities of interactions between Helmholtz members, their labs and the guests. Although “inviting people and taking care of all the mechanical parts” was the job of the club’s Secretary, everyone was helping in terms of making suggestions for speakers and topics.^12^ Initially, while his grant from the SDF was active, Crick reported that guest speakers at Helmholtz Club meetings were often invited researchers under his Visitors Program.^44^ Visiting scientists at the institutions represented by other club members were also invited as speakers. It seems that over time, the Helmholtz Club acquired quite a reputation despite its low-key semi-institutional existence: “It was fantastic. And everybody knew about it. To get an invitation to the Helmholtz Club … was a great honour for people all over the country, all over the world—to come to San Diego and speak in the Helmholtz Club.”^17^ David Hubel, who received the Nobel Prize in Physiology or Medicine in 1981 jointly with Torsten Wiesel for their work on information processing in the visual system, began the talk he gave in August 1985 by casting his eyes around the room and saying that it was perhaps the most distinguished audience that he had ever spoken to.^45^ Maybe as a consequence of the prestige that the club was acquiring, it apparently became something of a habit to use guests’ presence to the utmost, like having them give other talks, beside their Helmholtz Club lecture, at the various Californian institutions represented among regular club members—so that when Sejnowski took over as Secretary in 1988, writing to Crick to give him his assessment of “how best to continue the tradition”, one of his main points was to establish rules so as to limit abuse:

“… the problem of overworking the guest can be solved if one group is assigned as host, and everyone agrees that only the host can ask the guest to give a formal talk in addition to the Helmholtz Club meeting. This would not rule out informal visits to labs.”^46^

An incidental purpose of the Helmholtz Club was to play the role of recruiting ground. Thanks to the lengthy questioning and discussion of which the invited speaker was the focal point, “… the Helmholtz Club was the opportunity to really get sort of under the skin of the person, and find out what they were doing and why it was important.”^17^ Sejnowski pointed that the Helmholtz Club was never consciously used that way, but that it could happen, especially with junior people, postdocs whose work looked interesting but of whom not much was known yet. They would be invited to give a talk at a Helmholtz session, and it could lead to them being recruited at one or another of the institutions represented among the club members.^12^ Allman’s memory is that this process was nearly always done in a kind and supportive way; the rare testy exchanges involved speakers who were very well established scientists.^47^

Meanwhile, although work relationships and sometimes friendships pre-existed between many of its members, a major benefit of the Helmholtz Club was certainly to reinforce the building-up of inter-personal trust, by bringing its members together for day-long retreats on a regular basis, in a setting which allowed them to develop a degree of intimacy with each other’s ways of thinking and personalities. This, in turn, was bound to help interactions between its members multiply. Such interactions manifested themselves on many different levels. At the one-to-one, private end of the spectrum, individual researchers would be prone to share with one another early drafts of papers, preliminary experimental results, tentative experimental designs or theoretical gropings, seeking comments and opinions, often outside their own area of expertise, from fellow Helmholtz members who they trusted both for their scientific acumen and their discretion.^48^ At the institutional, public end of the spectrum, interactions could result in larger-scale collaborative ventures.

An important and long-lasting such collaborative venture, launched in 1986 and still going, run jointly by the Divisions of Biology, of Engineering and Applied Science, and of Physics, Mathematics and Astronomy at Caltech, was the Computation and Neural Systems (CNS) graduate programme.^49^ It was concocted between Hopfield, Mead (who may not yet have joined the Helmholtz Club when CNS started but did shortly afterward) and van Essen—and Allman,^15^ although he is not officially listed among the programme founders. Its aim was to concentrate “on the fascinating problems at the interface between cellular biology, neurobiology, electrical engineering, computer science, and physics.” (Computation and Neural Systems: A Birthday Bash to Celebrate the First 15 Years, pp. 28–29) As Mead once put it, it was “all about getting the neuroscientists and people doing the computer models and people into circuits all talking to each other” (Mead, 1996, pp. 21), and for him, in the early years of the CNS programme, “the Helmholtz Club was a very important connection for us, because it was a quite good illustration of how people in different fields could work together and collaborate … It was certainly an ‘existence proof’, you know, that people in really very, very different worldviews could actually get along and talk to each other.” Allman confirmed that there was a lot of overlap between CNS and the Helmholtz Club, and that many of the students and postdocs from the former were fairly regular attendees of the latter.^15^ There was also a lot of overlap between the CNS Faculty and the Helmholtz Club. Beside Hopfield, Mead, Allman and van Essen who created CNS, Christof Koch, Richard Andersen and current head of the programme Pietro Perona were regulars for periods of time; and Scott Fraser, Masakazu Konishi, Thanos Siapas and Doris Tsao were occasional attendees.^50^

In time, CNS spawned two distinct closely associated collaborative ventures at Caltech, expanding CNS scope as an interdisciplinary doctoral centre into further training and research. One was the National Science Foundation Engineering Research Center in Neuromorphic Systems Engineering (CNSE), started in 1995. It ran until 2006, with Pietro Perona as Director from 1999,^51^ which is well beyond the “five or seven years” of NSF-funding initially forecasted (Mead, 1996, p. 24). Mead, who was involved from the start, thus described the birth of the project:

“… we had a group of faculty who were in CNS and we were all working together. We shared grad students and we’d get together all the time. We’d have seminars and we’d go to dinner together. So a couple of the young faculty came around and said, ‘Hey, why don’t we get an NSF center?’ … And [CNSE], that’s different, because most NSF centers are shotgun marriages. You’ve got to gather up enough people to make it look like a center, but really they’re warring camps—and we didn’t have that, we already had a very successful academic program. We had these crossdisciplinary collaborations that were working already. We had a new art form that was developed quite far along, and people started applying it to real things.” (Mead, 1996, pp. 22–23)

Another collaborative venture, established in 1994, was Caltech’s Center for Theoretical Neurobiology, funded by the Alfred P. Sloan Foundation, which became a Sloan-Swartz Center for Theoretical Neurobiology after 2000 when the Swartz Foundation joined in. It was part of a group of five centres in the United States, which were meant to “serve as training and research exemplars … Each center would seek to closely couple an already established high quality experimental group with theorists. The theorists to be added—people trained as mathematicians, theoretical physicists or chemists, computer scientists—would be at graduate student, post-doc, or assistant professor level … The theorists would be expected to learn experimental procedures well enough so as to understand the capabilities, accuracy and limitations of experimental methods.” A few hand-picked university and research institutes were invited to submit proposals. Crick was among the scientists consulted by Hirsch Cohen, Program Officer in charge at the Sloan Foundation, to comment on the overall plan.^52^ His comments were, unsurprisingly, positive.^53^ Beside Caltech, the Salk Institute, UCSF, NYU and Brandeis University were chosen to house Centers for Theoretical Neurobiology. For Allman, the main reason why the Sloan Foundation picked Caltech was because they were interested in the training in theoretical-experimental crossovers between biology and computational modelling that the CNS programme had been offering for almost a decade, which was really one of its kind. As a result, CNS became the core of Caltech’s Sloan-Swartz Center: the Sloan and Swartz foundations funding enabled CNS to offer doctoral and post-doctoral fellowships to its students.^15^

It may be argued that the Helmholtz Club served yet another purpose, closely related to the role it played as ‘existence proof’ for the CNS programme and its evolutions. Either directly, because Helmholtz members would bring their postdocs and eventually doctoral students along, or indirectly, because they would carry back to their labs some intellectual seeds gathered at meetings, the club contributed to the enculturation of younger generations of researchers still in their formative years into a specific framing of neuroscience, which they would then help disseminate. It thus increased the chances of the research paradigm that the Helmholtz Club proposed for neuroscience to achieve future dominance. A case of long-ranging influence mediated by the Helmholtz Club was for instance that of Jack Gallant’s work in the 1990s, who was a postdoc in Van Essen Lab at Washington University School of Medicine where van Essen moved in 1992. In a letter to Crick of July 1994, van Essen wrote:

“… I wanted to mention a project that is just getting underway in my lab that was inspired in significant measure by a question you asked perhaps a decade or so ago at one of the Helmholtz meetings! As I recall it, your question was ‘How do neurons in visual cortex fire when a monkey is just looking around at a natural scene without any behavioral constraint?’ Neither I nor anyone present at that Helmholtz meeting knew the answer then, nor as far as I am aware does anyone else know the answer now in any substantial way.”^54^

Judging from the subsequent list of publications, this turned out to be a productive line of enquiry in Van Essen Lab.^55^

## 5 Epilogue

The Helmholtz Club was still in existence as of October 2012, but its meetings had become increasingly fewer and further apart—they went from being held monthly to quarterly to twice a year to about once a year these days according to the Secretary. Predictably, the issue is one of funding. The Salk Institute would not support it financially anymore and if it were to continue monthly, the club would have to get grant money from another source. Besides, as Sejnowski pointed out, there is a generational factor. After thirty years, some of the core members have died; many more have retired or are in the process of doing so and have thus dispersed. A new generation would have to take over for the Helmholtz Club to resume its former level of activity. Finally, looking back at the intent behind the creation of the Helmholtz Club in the early 1980s—to play for vision, then “in a state of confusion similar to pre-DNA molecular biology”, the role that the RNA Tie Club (and I would venture, the Hardy Club) had played for early molecular biology—while taking stock both of the degree to which visual neuroscience and cognitive neuroscience have become instituted, as well as of the prominence of South-Californian institutions in these fields, it may be that this particular semi-institutional gathering has fulfilled its seedbed role. The Helmholtz Club has certainly helped forge the link between cognitive neuroscience and visual neuroscience, further reinforcing the construction of the visual system as a model system for the study of consciousness and the higher cognitive functions more generally, alongside a set of experimental and computational techniques.

The present article had a dual aim in examining the history of the Helmholtz Club. The first, taking an individual-centric perspective, was to start filling the gaps in the seriously under-researched part of Francis Crick’s career that was spent at the Salk Institute doing neuroscience. As such, it has revealed the lasting influence that Francis Crick had over the Helmholtz Club: his patronage of the club, the place that it held in his plans for visual and cognitive neuroscience, and aspects of his indefectible commitment. And in view of the dedication that Francis Crick showed both to attending the Helmholtz Club and to keeping it afloat for over twenty years, it seems that his Californian years do indeed require scholarly attention if one considers that *Francis Crick: Hunter of Life*’*s Secret*, Crick’s landmark biography by Robert Olby, does not even mention the existence of the club (Olby, 2009).

The second aim of this article, focusing on the Helmholtz Club itself, was to vindicate the view that semi-institutional units of analysis are too easily overlooked by historians, by shedding light on the wide array of uses, cutting across the epistemological, social and political dimensions of science, that a semi-institutional gathering like a ‘mere’ scientific club could serve when skilfully organized, and thus on the part it could play as metaphorical greenhouse for a fledgling research programme to take root and eventually flourish. The Helmholtz Club was not alone in that respect but was indeed part of a wider configuration that worked towards the integration between various levels and different areas of scientific research on the mind-brain question, from the molecular to the systems, cognitive and behavioural levels. For instance, although its history remains to be written, it appears that an important US-based interdisciplinary forum for the institutionalisation of the neurosciences, on a bigger and much longer scale than the Helmholtz Club, was the Neurosciences Research Program (NRP). Started as an invisible college of thirty-four ‘gifted scientists’ by Francis O. Schmitt and a group of colleagues at MIT in 1961 when they had not yet coined the term ‘neuroscience’ (Schmitt, 1967, p. 562), it became a visible institution both more formal and larger than the Helmholtz Club, typically holding biannual week-long stated meetings of its associates alongside occasional themed conferences and workshops; but like the Helmholtz Club, it kept functioning on the co-optation mode, as new names were put forward and elected by the Active Associates to replace those who having finished their term were transferred to Honorary Associate status. Interestingly, the avowed aim of the NRP when it was first thought out was “to do for the brain what Watson and Crick had recently done for the gene” (Rose & Abi-Rached, 2013, p. 25). Schmitt’s vision for the NRP was to build a unified community out of disparate research fields belonging to the general areas of ‘molecular neurobiology’, ‘neural science’ and ‘behavioral or psychological science’ (Schmitt, 1967; Swazey, 1975). It is worth mentioning that Crick became an Active Associate of the NRP in the late 1970s, and remained an involved and regular attendant of its Stated Meetings even after his transfer to Honorary Associate status in 1989 at the end of his term, until 2003.^56^

Finally, running throughout and holding the paper’s dual aim together, is an invitation to broaden our understanding of what counts as ‘doing science’ and consider many kinds of activities beside laboratory work and intellectual elaborations, happening in a whole array of mundane places like canteens, pubs, musty seminar rooms, around coffee machines, walking across campuses, on planes to conferences—in an entire nondescript and often transient geography of science. Further, it is a prompt to reconsider definitions of success and failure in science: as revealed in the case of Crick’s contribution to the neurosciences, different understandings of what counts as ‘doing science’ can reconfigure what the received wisdom has construed as failure into success.

## Footnotes

1 For instance, Abir-Am (1987), Aronova (2012), de Chadarevian (1998, 2002), Gaudillière (1996), Kay (1993) and Kohler (1994).

2 See Frickel (2004), Frickel & Gross (2005), Frickel & Moore, Eds. (2006) and Moore (2008).

3 Especially McAdam (1989), Snow, Soule, & Kriesi (2004) and Tilly (2005).

4 I am indebted to an anonymous reviewer for pointing out in the first place that my account questions the definitions of success and failure in science.

5 Ridley (2006) and Olby (2009).

6 Wellcome Library, Francis Crick Papers, folder PP/CRI/D/1/5/14, letter Ramachandran to Crick, 22 April 1982; Ramachandran, personal communication, 24/01/2013; for more on the RNA Tie Club, see for instance Kay (2000) and Olby (2009).

7 Mandeville Special Collections Library, MSS 660, Francis Crick Personal Papers, Box 14, Appointment Book 1982.

8 Wellcome Library, Francis Crick Papers, folder PP/CRI/D/1/5/14, letters Crick to Ramachandran, 27 April 1982, and Ramachandran to Crick, 8 June 1982.

9 Wellcome Library, Francis Crick Papers, folder PP/CRI/D/1/4/17, letter Ramachandran to Crick, 18 December 1980.

10 Ramachandran, personal communication, 24/01/2013.

11 Sejnowski, personal communication, 05/10/2012; Ramachandran, personal communication, 24/01/2013.

12 Sejnowski, personal communication, 05/10/2012.

13 Mandeville Special Collections Library, MSS 660, Francis Crick Personal Papers, Box 14, Appointment Book 1982; Allman, personal communication, 27/09/2012.

14 Wellcome Library, Francis Crick Papers, folder PP/CRI/C/3/2, Report to System Development Foundation, 1 July 1982–30 June 1983, p. 3; I have traced six Helmholtz Club meetings beside the un-labelled one in September for the academic year 1982–83.

15 Allman and Mead, personal communication, 28/09/2012.

16 Churchland, personal communication, 05/10/2012.

17 Albright, personal communication, 02/10/2012.

18 Wellcome Library, Francis Crick Papers, folder PP/CRI/L/7/4, memorandum to ‘Helmholtz Club Members’, 3 April 1989, and handwritten notes, 18 April 1989.

19 Wellcome Library, Francis Crick Papers, folder PP/CRI/L/7/4, handwritten notes, 19 December 1989.

20 Allman and Mead, personal communication, 28/09/2012; there are several other sources confirming that Crick went religiously and almost never missed a meeting.

21 Wellcome Library, Francis Crick Papers, folder PP/CRI/C/7, Memorandum Sejnowski to Helmholtz Club Members.

22 Sejnowski, personal communication, 05/10/2012; Allman, personal communication, 21/05/2013.

23 Ramachandran, personal communication, 24/01/2013.

24 Allman, personal communication, 21/05/2013.

25 Wellcome Library, Francis Crick Papers, folder PP/CRI/C/3/1, Request to System Development Foundation, January 1982, p. 5.

26 Wellcome Library, Francis Crick Papers, folder PP/CRI/C/3/1, Request to System Development Foundation, January 1982, pp. 2–5.

27 David Hubel and Torsten Wiesel were neurophysiologists who from 1959 onward, through twenty-five years of collaboration spent for the most part at Harvard, did foundational work in vision neurophysiology, and in particular information processing in the visual system; they received the Nobel Prize in Physiology and Medicine in 1981.

28 Wellcome Library, Francis Crick Papers, folder PP/CRI/C/3/1, Draft letter de Hoffman to Smith, 15/01/1982.

29 Wellcome Library, Francis Crick Papers, folder PP/CRI/D/1/5/15, Letter Sejnowski to Crick, 05/07/1982.

30 Wellcome Library, Francis Crick Papers, folder PP/CRI/C/3/2, Report to System Development Foundation, 1 July 1982–30 June 1983, p. 3.

31 Wellcome Library, Francis Crick Papers, folder PP/CRI/C/3/2; Mandeville Special Collections Library, MSS 660, Francis Crick Personal Papers, Box 14, Appointment Books 1982, 1983, 1984, 1985; the record of Helmholtz Club speakers for the early years is very patchy.

32 Wellcome Library, Francis Crick Papers, folder PP/CRI/C/3/2, Report to System Development Foundation, 1 July 1983–30 June 1984, p. 3.

33 Wellcome Library, Francis Crick Papers, folder PP/CRI/C/3/2.

34 Wellcome Library, Francis Crick Papers, folder PP/CRI/J/1/2/15, letter Sejnowski to Crick, 3 August 1988.

35 Crick (1966) and Watson (2007).

36 A perusal of his Appointment Books (Mandeville Special Collections Library, MSS 660, Francis Crick Personal Papers, Box 14) is indeed enough to ascertain this fact.

37 PDP stands for Parallel Development Processing; the meetings were discontinued when their organiser David Rumelhart moved to UCLA in 1987; the work and regular meetings of the PDP group led to the publication of McClelland & Rumelhart (1986), the influential 2-volume ‘PDP Bible’ as it came to be known in Artificial Intelligence circles, which put connectionist AI back in fashion while symbolic AI became for many GOFAI, Good Old Fashioned Artificial Intelligence: see Boden (2006, chap. 12).

38 Churchland, personal communication, 05/10/2012.

39 Mandeville Special Collections Library, MSS 660, Francis Crick Personal Papers, Box 3, Folder 12, Δ^2^V Club, undated, and Box 4, Folder 37, Kapitza Club, 1951–57.

40 Allman recalls that he was the ‘local’ speaker at this first official Helmholtz meeting; his talk involved a demonstration of the non-classical receptive field effect that required a specially constructed computer to generate the images and his research engineer, Francis Miezin, came along to make sure it worked (Allman, personal communication, 21/05/2013).

41 This was confirmed by several Helmholtz members (Sejnowski, Mead and Allman, Albright).

42 Sejnowski, personal communication, 05/10/2012; Allman and Mead, personal communication, 28/09/2012; Albright, personal communication, 02/10/2012.

43 I rely on the concept of trust as property of inter-personal relations as developed by sociological historian of political movements Charles Tilly in Tilly (2005).

44 Wellcome Library, Francis Crick Papers, folder PP/CRI/C/3/2, Report to System Development Foundation, 1 July 1982–30 June 1983, p. 3; I have been able to confirm that it was indeed the case, for that year and subsequent years until 1985, by matching Crick’s annual lists of visitors with speakers at Helmholtz Club meetings confirmed through other sources.

45 Allman, personal communication, 21/05/2013.

46 Wellcome Library, Francis Crick Papers, folder PP/CRI/J/1/2/15, letter Sejnowski to Crick, 3 August 1988.

47 Allman, personal communication, 21/05/2013.

48 Francis Crick Papers at the Wellcome Library abound with such instances; see for example, PP/D/1/5/7, letters between Crick and Hopfield, November 1982–March 1983, PP/CRI/J/1/1/1/1, letters between Crick and Allman, March–April 1984, PP/CRI/J/1/1/11/1, letters between Crick and MacLeod, February–March 1985, PP/CRI/J/1/3/14/1, letters between Crick and Ramachandran, March–November 1990; other such instances transpire in the acknowledgement sections of published papers, for example Ramachandran & Cavanagh (1985).

49 http://www.cns.caltech.edu/index.html, consulted 08/05/2013.

50 Current CNS Faculty is listed at http://www.cns.caltech.edu/people/index.html, consulted 08/05/2013.

51 According to Pietro Perona’s résumé, http://www.vision.caltech.edu/html-files/perona-resume.html, retrieved 10/05/2013.

52 Wellcome Library, Francis Crick Papers, folder PP/CRI/J/1/4/17/1, letters Crick to Cohen, 23/02/1994, and Cohen to Crick, 22/03/1994.

53 Wellcome Library, Francis Crick Papers, folder PP/CRI/J/1/4/17/1, proposed Sloan Program in Theoretical Neurobiology, January 1994.

54 Wellcome Library, Francis Crick Papers, folder PP/CRI/J/1/4/19, letter van Essen to Crick, 13/07/1994.

55 Van Essen Lab: Publications, http://brainvis.wustl.edu/wiki/index.php/Lab: Publications, consulted 27/03/2013.

56 Wellcome Library, Francis Crick Papers (PP/CRI): there are many folders related to Crick’s involvement with the NRP, containing some correspondence as well as programmes for the Stated Meetings of the NRP Associates that he attended regularly from the time he became an Active Associate in 1978 until the 2000s. For more on the NRP, see Schmitt (1967), Swazey (1975) and Rose & Abi-Rached (2013, chap. 1).
